# Acceptor Side-Chain Effects on the Excited State Dynamics of Two-Dimensional-Like Conjugated Copolymers in Solution

**DOI:** 10.3390/molecules22091398

**Published:** 2017-08-25

**Authors:** Ming-Ming Huo, Rong Hu, Wei Yan, Yi-Tong Wang, Kuan W. A. Chee, Yong Wang, Jian-Ping Zhang

**Affiliations:** 1 Qingdao Research Center for Advanced Photonic Technologies, Laser Research Institute, Shandong Academy of Sciences, Qingdao 266100, China; weiyan@sdlaser.cn (W.Y.); wythyx@163.com (Y.-T.W.); kuan.chee@nottingham.edu.cn (K.W.A.C.); yongwang@sdlaser.cn (Y.W.); 2 Research Institute for New Materials Technology, Chongqing University of Arts and Sciences, Chongqing 402160, China; hurong_82@163.com; 3 Department of Electrical and Electronic Engineering, Faculty of Science and Engineering, University of Nottingham Ningbo China, Ningbo 315100, China; 4 Department of Chemistry, Renmin University of China, Beijing 100872, China; jpzhang@chem.ruc.edu.cn

**Keywords:** two-dimensional like conjugated copolymers, acceptor side-chain, transient absorption spectrum, intramolecular charge-transfer characters, excited state dynamics

## Abstract

Excited state dynamics of two-dimensional-like conjugated copolymers PFDCN and PFSDCN based on alternating fluorene and triphenylamine main chains and malononitrile pendant acceptor groups with thiophene as π-bridge, have been investigated by using transient absorption spectroscopy. There is an additional conjugated –C=C– bond in PFDCN, which distinguishes it from PFSDCN. The lowest energy absorption band of each copolymer absorption spectrum is attributed to the π−π* transition with intramolecular charge-transfer, which has a lower fluorescence contribution than those of higher energy absorption bands. The optical excitation of either PFDCN or PFSDCN solution generates polaron pairs that then self-localize and evolve to a bound singlet exciton within a few picoseconds. Due to the additional conjugated –C=C– bond in the acceptor side-chain, PFDCN has a stronger intramolecular charge-transfer characteristic compared with PFSDCN, therefore exhibiting a longer self-localization time (7 ps vs. 3 ps for PFSDCN) and a shorter fluorescence lifetime (1.48 ns vs. 1.60 ns for PFSDCN).

## 1. Introduction

π-Conjugated polymers have applications in a range of devices such as organic light emitting diodes (OLEDs), field effect transistors, and solar cells [1]. The photo excitation properties in these materials have been extensively studied by experiment and theory. In sharp contrast with inorganic semiconductors, the general photo excitation products found in polymers are tightly bound excitons. To enable the use of such polymers in solar cells, they are normally mixed with electron-accepting materials (such as [6,6]-phenyl-C_61_-butyric acid methyl ester (PCBM)) to form a bulk-heterojunction (BHJ) i.e., donor-acceptor (D-A) junction providing the necessary driving force for the dissociation of photogeneration products. Recently, BHJ organic solar cells based on polymers and fullerenes have achieved a light-to-electric power conversion efficiency (PCE) of 10% [2,3,4,5]. However, the primary products of photoexcitation in polymer solutions and neat films have long been controversial. Photogeneration products are the result of local excitation known as Frenkel exciton [6,7] or interband transition which directly produce free carriers [8,9], with the primary type being the main point of controversy. Some researchers considered that optical excitation also creates a wealth of other quasiparticles, polaron pairs, biexcitons and others [10,11]. All of the above controversies are concerned with homopolymer systems, such as poly(3-hexylthiophene) (P3HT), poly(phenylene-vinylene) (PPV), etc. Recently, many research results are focused on the photogeneration of charged species, for instance, De Sio et al. used ultrafast two-dimensional electronic spectroscopy to study the dynamics of polaron pairs formation in rr-P3HT thin film on a sub-20-fs time scale indicating that coherent vibronic coupling induces ultrafast polaron pair formation [12].

Molecular engineering is one of the most widely used methods to improve the performance of polymers. In contrast with homopolymers, the absorbance and energy levels of D-A copolymers can be tuned by controlling the intramolecular charge transfer (ICT) from the donor to the acceptor units along their conjugated backbone. So far, polymer solar cells (PSCs) based on linear D-A copolymers have achieved the best PCE [5]. In linear D-A copolymer systems, abundant evidence indicates that optical excitation creates ultrafast charge separation before localization of the primary excitation to form a bound exciton in solution and neat films [13,14,15,16]. For instance, Heeger et al. investigated PCDTBT chlorobenzene solutions and thin films by femtosecond-resolved fluorescence up-conversion spectroscopy and concluded that mobile electrons and holes were photogenerated by interband π−π* transitions, which then spatially self-localize and bind within an exciton in less than 1 ps [13]. Their following research revealed that the photoexcitation process generates delocalized coherent superposition states, which is a natural consequence of the Heisenberg uncertainty principle [15]. These delocalized coherent superposition states can be defined as polaron pairs, which are also called charge-transfer excitons, in which spatially separated electrons and holes weakly interact via their Coulombic attraction. Our group earlier investigated PBDTTTs solutions [17,18], photogeneration of the ratio of CS state, charge-transfer (CT) state and the *S*_1_-exciton dependence on the excitation energy and side chain, which mainly affect the chain conformation of the PBDTTTs in solution.

However, the linear D-A copolymers with alternating electron-rich (donor) and electron-deficient (acceptor) units along their conjugated backbone, the molecular interactions and packing orientation of the conjugating moieties need to be carefully controlled to ensure proper processability and charge transporting [19,20]. This may increase the difficulty of molecular control and design. Different from linear D-A copolymers, two-dimensional (2D) like D-A copolymers have been designed and synthesized in recent years with the strong electron-withdrawing acceptor group structures on the ends of the side chains and connecting with the electron-donating conjugated main chain through a π-bridge [21,22,23]. In this way, the bandgaps of the resulting D-A polymers can be effectively tuned by changing the pendant acceptor groups on the side chains, while the relatively deep highest occupied molecular orbital (HOMO) energy levels can be maintained by using electron-donating units to build the main chains. However, so far very little research has been done on the excited state properties of this kind of copolymers. Although the PCE of the device based on 2D=like D-A copolymers is lower than that based on linear D-A copolymers, it more or less mirrors the level of P3HT devices, hence the inherent properties of the 2D like copolymers are very promising, and are yet unexplored.

In the present work, the excited state dynamics of 2D=like conjugated PFDCN and PFSDCN copolymer solutions have been detected by using the corresponding femtosecond time-resolved absorption spectra. The purpose of the work was to study the effects of the side chain on the excited dynamics of PF(S)DCN solutions. It is directly observed that the primary photoexcitation products in PF(S)DCN solutions are polaron pairs, which then self-localize and evolve to bound singlet exciton states within a few picoseconds. The self-localization time is dependent on the acceptor group side chains.

## 2. Results and Discussion

### 2.1. Steady-State Spectroscopies

**Figure 1:**
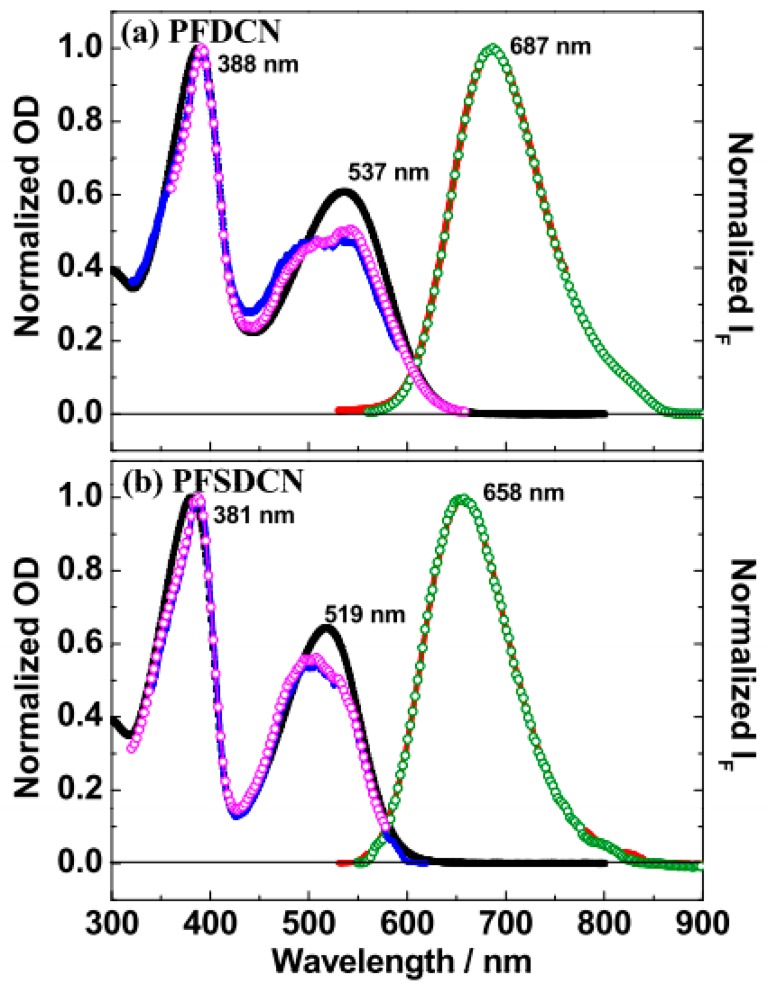
Steady-state spectra of PFDCN (**a**) and PFSDCN (**b**) in chlorobenzene solution. The absorption spectra (black line), The fluorescence spectra with 390 nm and 540 nm excitation for PFDCN, 380 nm and 520 nm excitation for PFSDCN (red line and green line), the fluorescence excitation spectra probed at 610 nm and 690 nm (blue and magenta).

The two apparent featureless absorption peaks can be seen at 388 nm and 537 nm for a PFDCN chlorobenzene solution (Figure 1a), and at 381 nm and 519 nm for the corresponding PFSDCN solution (Figure 1b), respectively.

The peak at 388 nm for PFDCN (or 381 nm for PFSDCN) corresponds to the π−π* transition of the PFDCN (PFSDCN) conjugated main chains [24,25]; while the absorption peak at 537 nm for PFDCN (519 nm for PFSDCN) corresponds to the π−π* transitions with intramolecular charge transfer (ICT) characteristics, i.e., shift of electron densities upon optical excitation from the main chains to the side chains [21,25]. The ICT absorption band is the lowest absorption state (*S*_1_), where the electrons and holes still strongly interact via their Coulombic attraction. Similar spectral features were reported for other D-A copolymers like poly([2,7-(9,9-bis(3,7-dimethyloctyl)fluorene)]-*alt*-[5,5-(4,7-di-2′-thienyl-2,1,3-benzothiadiazole)]) (PFDTBT) and poly[*N*-11″-henicosanyl-2,7-carbazole-*alt*-5,5-(4′,7′-di-2-thienyl-2′,1′,3′-benzothiadiazole)]) (PCDTBT) [13,25]. Compared with PFSDCN, the ICT absorption band of PFDCN is red-shifted by 18 nm and broadened by 800 cm^−1^, which means an additional conjugated –C=C– bond in the side-chain of PFDCN has stronger ICT characteristics. Here, some absorption bandx may reflect polymer aggregation in solution [26]. In the PF(S)DCN solution system, polymer aggregation has been ignored. First, we compared the ground-state absorption of the polymer solutions over a range of monomer-based concentrations (e.g., for PFDCN solutions, the concentration ranged from 2.3 × 10^−6^ M to 2.7 × 10^−5^ M, see Supplementary Material Figure S1). The nearly identical spectra obtained upon varying the concentration by two orders of magnitude suggest that there is no intermolecular aggregation in the polymer solution; second, the structure of the malononitrile pendant acceptor groups with thiophene as π-bridge is not conducive to self-aggregated polymers. Besides, many other polyfluorene copolymers (Dio-PFDTBT, F8BT) solution show similar ‘‘camel-back’’ absorption spectra as PFDCN or PFSDCN, while also having no aggregation.

A single unstructured emission peak can be seen at 687 nm for the PFDCN solution (red curve and green circles in Figure 1a, and at 658 nm for the PFSDCN solution (Figure 1b). Compared to PFSDCN, a lower excited state energy is indicated in PFDCN. The Stokes shift, given by the energy difference between the ICT absorption peak and the emission peak, is ~4070 cm^−1^ (0.51 eV) in the two copolymers. All relaxation processes after photoexcitation contribute to the Stokes shift, including thermalization of the electrons and holes to the edge of the conduction and valence band, respectively, vibrational relaxation, localization of the excitation, exciton formation, exciton migration, and structural relaxation [13]. Besides the Stokes red-shift, the emission bands of both PFDCN or PFSDCN are narrower than the ICT absorption band, respectively. This suggests a higher coplanar conjugate skeleton in excited state of copolymers than that in the ground state.

The fluorescence excitation spectra of the pair of copolymers are obtained by probing the fluorescence near the band blue edge (610 nm) and maximum (690 nm) (blue curve and magenta circles in Figure 1a,b). Deviating slightly from their respective absorption spectra, the fluorescence excitation spectra show a lower ICT band, which is due to lower emission efficiency of the ICT absorption band. This lower emission efficiency can be explained by the ICT characteristics of the copolymer side-chain, which to some extent reduces the overlap between electrons and holes, leading to a decreased Coulombic attraction and lower binding energy. Finally, the fluorescence quantum yields of the two copolymers are consistent, being 0.11 ± 0.02. The molar extinction coefficient of the ICT absorption band has been measured where for PFDCN at 537 nm is 4.96 × 10^5^ L·mol^−1^·cm^−1^, and for PFSDCN at 519 nm is 2.06 × 10^6^ L·mol^−1^·cm^−1^.

### 2.2. Time-Resolved Absorption Spectroscopies

#### 2.2.1. PFDCN in Chlorobenzene

**Figure 2:**
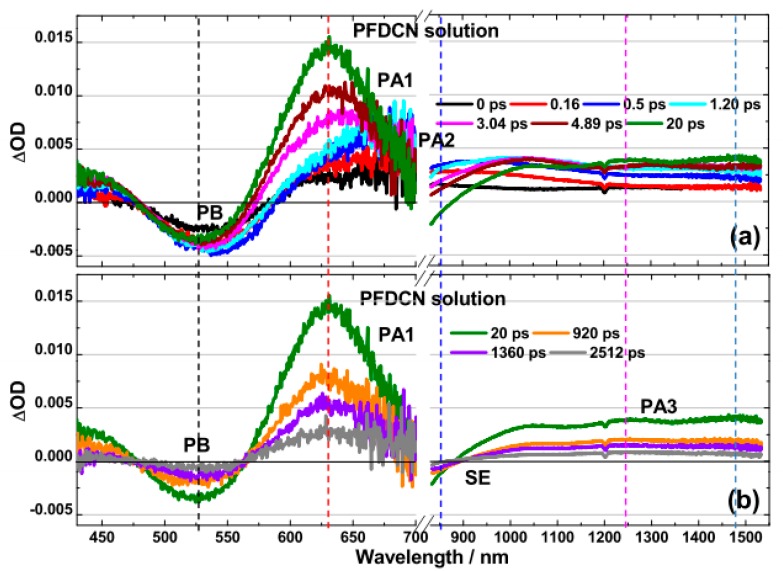
Transient absorption spectra of PFDCN in chlorobenzene solution with excitation wavelength at 400 nm (6.30 × 10^13^ photons·cm^−2^·pulse^−1^). (**a**) Spectra at first 20 ps and (**b**) spectra at 20 ps–2512 ps in visible and NIR region. The break (/ /) from 700 nm to 830 nm, where be interference by laser at 800 nm.

The visible and near-infrared TA spectra of PFDCN in chlorobenzene solution photoexcited at 400 nm are shown in Figure 2. It is seen from Figure 2a that the TA spectra around 530 nm exhibits a negative feature, which is consistent with the ICT absorption band (Figure 1), coming from ground state photobleaching (PB). There immediately follows the pulsed optical excitation (0.0 ps, 0.16 ps). Instead of presenting a negative *S*_1_ → *S*_0_ stimulated emission (SE), the region from 600 nm to 850 nm shows a broad positive absorption band (PA2). The PA2 gradually decreases during the following 20 ps, in the meantime, a new absorption band PA1 (around 630 nm) gradually rises and the negative *S*_1_ → *S*_0_ stimulated emission appears in the PA2 region. Interestingly, the absorption at 1200 nm~1550 nm (PA3) and PA1 increases at the same rate in the first 20 ps. However, different from PA1, the ascent of PA3 is accompanied by a blue-shift, with dual absorption features around 1250 nm and 1480 nm respectively, which are discernible till ~20 ps. Visible and near-infrared TA spectra of PFDCN in chlorobenzene solution in longer delay time >20 ps are shown in Figure 2b. Following the long delay, there are no spectral and isoabsorptive point (~563 nm) shifts and all transient features (PB, SE, PA1 and PA3) decay at the same rate. It means that no other new species have appeared during the examined period of time.

PA1 is originated from the *S*_1_ → *S*_n_ singlet exciton for its decay time well in consistent with the fluorescence kinetics (see Supplementary Material Figure S2). From 20 ps to 2.5 ns, PA3 is in accordance with the decay of PA1 and SE, so PA3 is originated from the absorption of singlet exciton up to other energy level *S*_1_ → *S*_m_ (the energy level of *S*_m_ is lower than that of *S*_n_), where 950 nm, 1250 nm and 1480 nm are the three vibration bands of *S*_m_.

**Figure 3:**
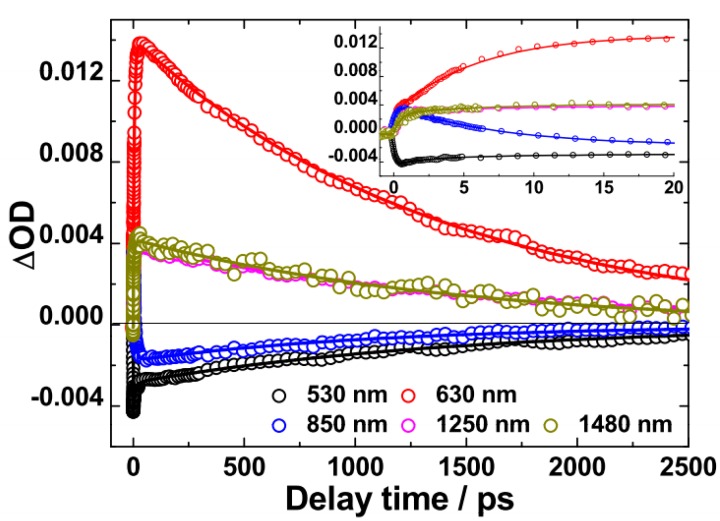
Kinetics at the indicated probing wavelengths plotted from the corresponding spectral dynamic in Figure 2 for PFDCN in chlorobenzene solution, Solid lines are fitting curves based on multi-exponential model functions.

Based on our observations of the above TA spectra, the dynamics at λ = 530 nm (PB), 630 nm (*S*_1_ → *S*_n_), 1250 nm and 1480 nm (*S*_1_ → *S*_m_), 850 nm (where PA2 and SE are overlapped) have been further investigated into, as shown in Figure 3.

The decay time constants are listed in Table 1. The 630 nm kinetics exhibit a slow rise phase (τ_2_ ~ 6.99 ps) prior to a monotonic decay (τ_3_ ~ 1.48 ns). The rise phase of 630 nm is in accordance with the decay of 850 nm (τ_2_ ~ 7.42 ps). Then, the 850 nm exhibits obvious SE characteristics with the decay time constant being ~1.44 ns. Such picosecond rise-to-decay correlations between the 630 nm and the 850 nm kinetics are strongly indicative of the process of PA2 → PA1 conversion. The PA2 band is originated from the absorption of polaron pairs, because dynamic decay of PA2 has an independent correlation with excitation fluences (see Supplementary Material Figure S3) [27,28]. Different from polaron pairs in polymer blend films, the polaron pairs consist of the radical cation of the donor and the radical anion of the acceptor. These polaron pairs are delocalized coherent superposition states, which are also called charge-transfer excitons, in which spatially separated electrons and holes weakly interact with each other via their Coulombic attraction [15,27]. They are an intermediate state.

#### 2.2.2. PFSDCN in Chlorobenzene

**Figure 4:**
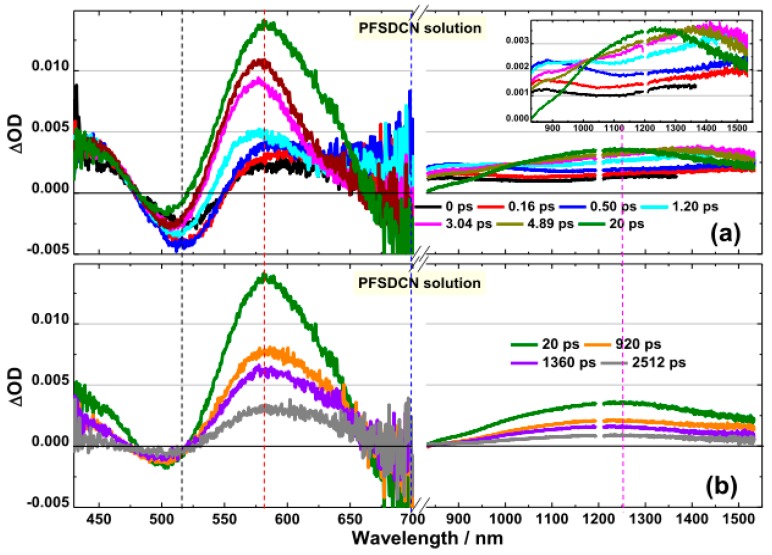
Transient absorption spectra of PFSDCN in chlorobenzene solution with excitation wavelength at 400 nm (6.30 × 10^13^ photons·cm^−2^·pulse^−1^). (**a**) Spectra at first 20 ps and (**b**) spectra at 20 ps–2512 ps in visible and NIR region. The break (/ /) from 700 nm to 830 nm, where be interference by laser at 800 nm. The *y*-axis be amplified in NIR region is shown in the inset for obviously observe the changing trend.

The visible and near-infrared TA spectra of PFSDCN in chlorobenzene solution photoexcited at 400 nm are shown in Figure 4. Like PFDCN, the TA spectra for PFSDCN around 500 nm exhibits a negative feature, also coming from ground state photobleaching. It is seen from Figure 4a that around 850 nm, there is a broad positive absorption band immediately following the pulsed optical excitation (0.0 ps, 0.16 ps), which comes from the absorption of polaron pairs.

Absorption of polaron pairs gradually decreases in the following 20 ps, and a new absorption band around 580 nm is gradually generated from the absorption of *S*_1_ → *S*_n_ singlet exciton. The absorption of longer wavelength at 1100 nm–1550 nm originated from singlet exciton up to other electronic energy level *S*_1_ → *S*_m_, which shows a wide absorption peak at 1250 nm till ~20 ps. Visible and near-infrared TA spectra of PFSDCN in chlorobenzene solution with longer delay times >20 ps have been shown in Figure 4b. Within the long time, there are no spectral and isoabsorptive point (~519 nm) shifts and all transient features decay at the same rate. No other new species have appeared during the period of time.

**Figure 5:**
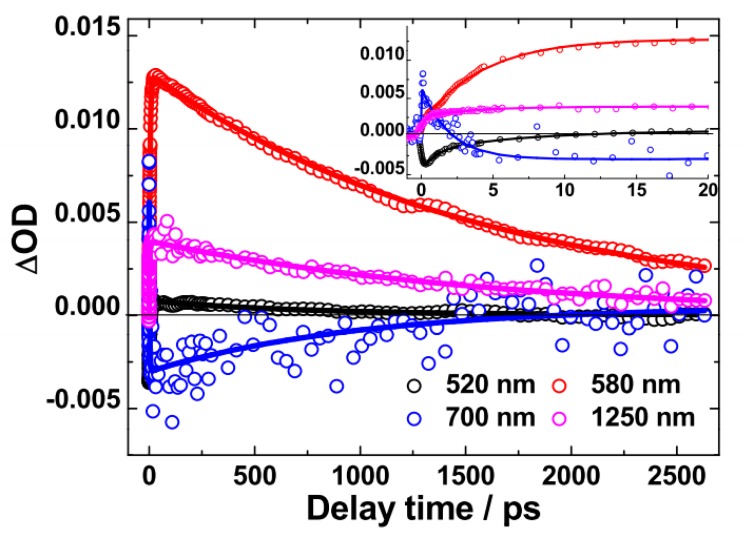
Kinetics at the indicated probing wavelengths plotted from the corresponding spectral dynamic in Figure 4 for PFSDCN in chlorobenzene solution, Solid lines are fitting curves based on multi-exponential model functions.

Based on above observations of the PFSDCN TA spectra, the dynamics of PB (520 nm), *S*_1_ → *S*_n_ singlet exciton (580 nm), polaron pairs and SE (700 nm), singlet exciton up to *S*_1_ → *S*_m_ (1250 nm) have been investigated and illustrated in Figure 5. Their decay time constants are listed in Table 2. The 580 nm kinetics exhibit a slow rise phase (τ_1_ ~ 3.30 ps) prior to a monotonic decay (τ_2_ ~ 1.60 ns). The rise phase of 580 nm is in accordance with the decay of 700 nm (τ_2_ ~ 2.81 ps). Then, the 700 nm exhibits obvious SE characteristics with the decay time constant being ~1.50 ns. Similar as PFDCN, such picosecond rise-to-decay correlation between the 580 nm and the 700 nm kinetics is strongly indicative of the process of polaron pairs → singlet exciton (*S*_1_ → *S*_n_, *S*_m_) conversion. Different from PFDCN, the conversion time from polaron pairs to singlet exciton (*S*_1_ → *S*_n_, *S*_m_) in PFDSCN is shorter (7 ps vs. 3 ps, respectively). In addition, the decay time of PB from 0 to 20 ps suggests that a proportion of polaron pairs has recombined to the ground states in the PFSDCN solution, which will be analyzed in the following context.

**Figure 6:**
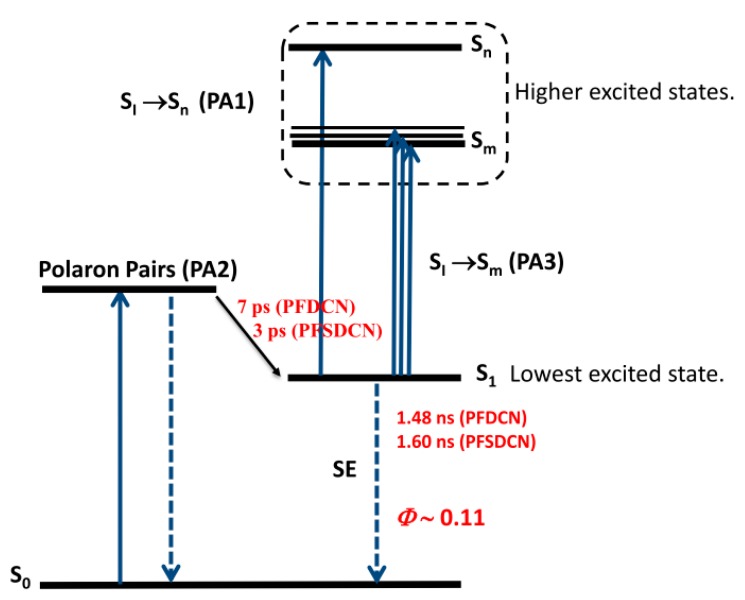
Energy diagrams for PF(S)DCN in chlorobenzene solution. The photo excitation produce mainly is polaron pairs, which geminate recombination to singlet exciton in 3–7 ps.

Based on the above analysis in Section 2.2.1 and Section 2.2.2, we proposed the energy diagram for the excited state in PFDCN and PFSDCN solution shown in Figure 6. Polaron pairs are generated by photoexcitation in 2D like D-A copolymer PFDCN or PFSDCN solution and through geminate recombinations become singlet excitons in the following 7 ps or 3 ps, respectively, after which the singlet excitons may transit to higher levels (*S*_1_ → *S*_m_, *S*_1_ → *S*_n_) or radiate to the ground state in their lifetime ~1.48 ns or 1.60 ns. It is worth noting that the high energy absorption band in Figure 1 was excited (400 nm) in fs-TA spectra. However, the TA spectra from 430 nm to 1550 nm in Figure 2 and Figure 4 do not show the absorption of *S*_2_ or hot *S*_1_ like other polymers [29,30]. We speculate the “π−π*” state (*S*_2_ or hot *S*_1_) may dissociate into polaron pairs within 0.16 ps because of the large charger-transfer characteristics of the 2D copolymers. When the ICT band in Figure 1 and the bandgap of the polymer is excitated (505 nm and 600 nm), polaron pairs are also generated by photoexcitation. (to PFDCN as an example, supplementary material Figure S4). The “π−π*” state is not shown in Figure 6 as it needs to be proved by other experiments with higher temporal resolution. Besides, some special relaxation pathways e.g., conformation relaxation may also exist. This photogeneration mechanism is different from other linear copolymers and homopolymers. For instance, in poly[4,8-bis-substituted-benzo[1,2-*b*:4,5-*b*′]dithiophene-2,6-diyl-*alt*-4-substituted-thieno[3,4-*b*]thiophene-2,6-diyl] (PBDTTT) solution systems, photogeneration of the singlet exciton, ICT and charge state appear simultaneously with different proportions and ICT recombine to ground state or dissociate to charge state depending on side chain functional groups, solution properties and excitation energy [17,18]. In P3HT solution systems [31], photogeneration of singlet exciton with a characteristic lifetime of 600 ps has been indicated, with P3HT triplet state formed by intersystem crossing from the singlet state with a lifetime of around 300 ns. The different excitation characteristics are from their special structural features of various polymers.

**Figure 7:**
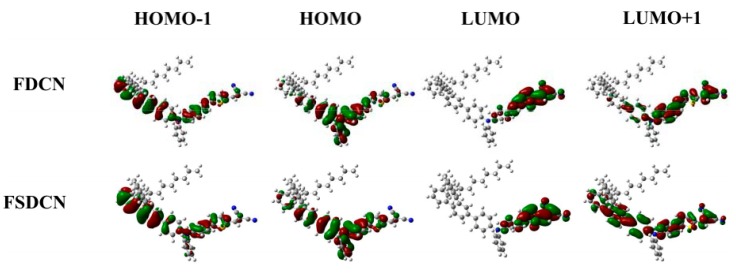
Plots of electron density of the indicated molecular orbitals for FDCN and FSDCN monomer. Geometry optimization was performed with Gaussian 09 package employing ONIOM modeling. The conjugated systems were optimized using B3LYP density functional at 6–31G (d,p) level, whereas the n-octyl side chains were treated semi-empirically using PM6 method. The single point energies were precisely calculated using B3LYP density functional at 6–311++G (2d,2p) level.

The 2D-like D-A copolymers have a structure with the strong electron-with-drawing acceptor groups on the end of the side chains and connecting with the electron-donating conjugated main chain through a π-bridge, and this construction may lower Coulombic interactions between electrons and holes and increase the likelihood of photogeneration of the polaron pairs. The difference in electron density between the ground state and excited state of FDCN and FSDCN monomer is shown in Figure 7 [31]. The larger delocalization of electron clouds have strongly proved the above analysis. Interestingly, the photogeneration of the polaron pairs have not recombined to ground state or dissociated to charge state within their lifetime, but have recombined to a singlet exciton. The phenomenon may be due to special construction of 2D D-A copolymers, which will be explained in detail below. Slow geminate recombination from polaron pairs to singlet excitons occurs only in the copolymer solution. When the interchain interaction is introduced in copolymer film, the geminate recombination time of polaron pairs will be shortened (<0.16 s) (Supplementary Material Figure S5).

### 2.3. Effect of Side Chains

**Figure 8:**
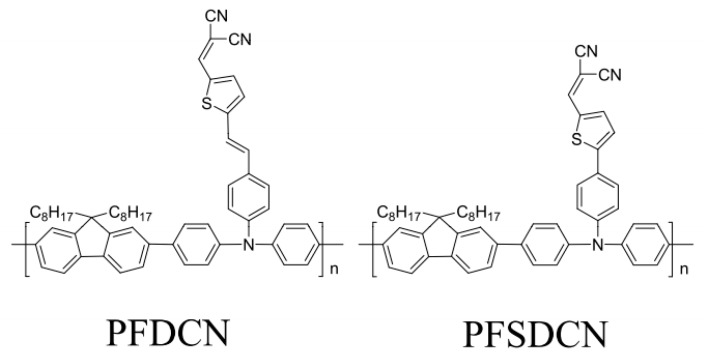
Chemical structures of PFDCN and PFSDCN.

The photoactive layers in PSCs are cast from the precursor solutions of polymers or polymer/fullerene mixtures, so it is expected that the knowledge of the side-chain effects on the molecular conformations and excited state properties of solution-phase polymers will facilitate an understanding of how side-chain substitution eventually affects device performance. What makes PFDCN and PFSDCN different is that the former has one more conjugated –C=C– bond in its side chain than the latter, as shown in Figure 8, PFDCN and PFSDCN have the same HOMO energy level (−5.3 eV) and open-circuit voltages *V*_OC_ (0.99 eV), but their most optimized PSCs devices perform differently from each other [21,25]. From PFSDCN to PFDCN, the LUMO energy level reduces from −3.27 eV to −3.43 eV, so the band gap (*E*g) reduces from 2.05 eV to 1.87 eV, the short-circuit current (*J*_SC_) increases from 5.42 mA/cm^2^ to 9.62 mA/cm^2^, hole mobility (μ) from 1.29 × 10^−4^ cm^2^·V^−^·s^−1^ to 5.27 × 10^−4^ cm^2^·V^−1^·s^−1^, Fill Factor (*FF*) from 40% to 50% and the PCE from 1.6% to 4.5% [21,25].

**Figure 9:**
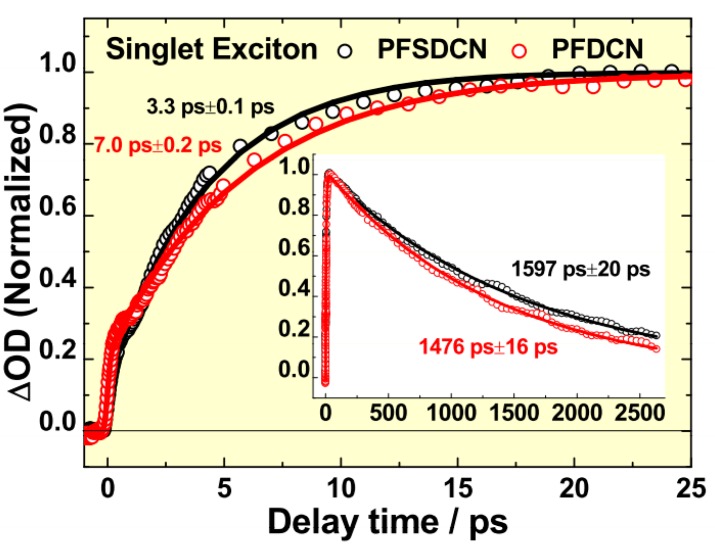
Comparison of *S*_1_ → *S*_n_ singlet exciton arising kinetics of PFDCN (red) and PFSDCN (black) solution. Insert shows the kinetics up to 2.5 ns for clearer view of the decay process. Solid lines are fitting curves based on multi-exponential model functions.

The differences between PFDCN and PFSDCN in terms of fluorescence lifetimes and geminate recombination time of polaron pairs to singlet exciton have been shown in Figure 9. The slow rise phase of *S*_1_ → *S*_n_ singlet exciton of the two copolymers is from the decay of their respective polaron pairs, 7.0 ps vs. 3.3 ps for PFDCN and PFSDCN, respectively. The additional conjugated –C=C– bond in PFDCN lengthens the conversion time from polaron pairs to *S*_1_ → *S*_n_ singlet exciton. This phenomenon means that the photogeneration of polaron pairs of PFDCN has an average longer electron-hole distance and hence reduced Coulombic attraction, which may facilitate charge separation, when they are spin-coated into neat films or photoactive layers with fullerene. The Insertion of Figure 9 compares the decay process (lifetime) of *S*_1_ → *S*_n_ singlet exciton of PFDCN with that of PFSDCN (1.48 ns vs. 1.60 ns). The additional conjugated –C=C– bond makes the exciton lifetime of PFDCN shorter than that of PFSDCN by ~120 ps. Although there is no charge carrier production in the two 2D-like copolymer solutions, the shorter exciton lifetime also suggests stronger charge transfer characteristics for PFDCN excitons, which may allow a higher dissociation efficiency.

**Figure 10:**
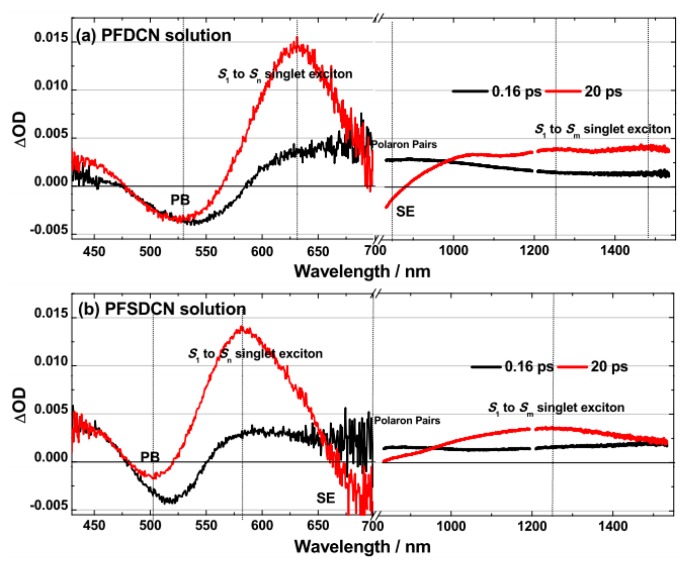
Comparison of transient absorption spectra of PFDCN (**a**) and PFSDCN (**b**) at 0.16 ps and 20 ps delay time.

According to the above analysis, there are no other new species appearing in the transient absorption spectrum of the two 2D like copolymers in the longer delay time >20 ps. (Figure 2 and Figure 4), so by comparing the ∆OD of *S*_1_ → *S*_n_ singlet exciton to PB at 20 ps, the molar extinction coefficient of *S*_1_ → *S*_n_ singlet exciton (ε_ex_) can be obtained, as shown in Figure 10.

ε_ex_ of PFDCN is 2.33 × 10^6^ L·mol^−1^·cm^−1^ (at 630 nm), while ε_ex_ of PFSDCN is 1.40 × 10^7^ L·mol^−1^·cm^−1^ (at 580 nm). Besides, comparing PB (530 nm) of 0 ps with 20 ps in PFDCN (Figure 10a), no obvious decay has been shown, which means no transient species have returned to ground states in this period. Therefore the conversion rate from polaron pairs to singlet excitons is close to 100%. However, in PFSDCN, the ∆OD of PB (500 nm) has reduced by 46% from 0 ps to 20 ps (Figure 10b), meaning only 54% polaron pairs converted into singlet excitons, while others have recombined to the ground state. The proportion of polaron pairs, which quickly recombine to the ground states in PFSDCN, may cause the waste of incident photon, inducing the lower *J*_SC_ when they are spin-coated into photoactive layers with fullerene.

## 3. Materials and Methods

The 2D-like conjugated copolymers PFDCN (M_w_ = 17.7 kg/mol, M_w_/M_n_ = 1.7) and PFSDCN (M_w_ = 37.5 kg/mol, M_w_/M_n_ = 2.0) shown in Figure 8 were synthesized following [21] and [32]. PFDCN and PFSDCN consist of an alternating fluorene and triphenylamine main chain, and malononitrile pendant acceptor groups with thiophene as π-bridge, There is an additional conjugated –C=C– bond in PFDCN, which distinguishes it from PFSDCN (Figure 8). Both of the copolymers exhibit similar HOMO energy (−5.3 eV) levels due to their same donor main chain, while their LUMO energy levels and optical band gap are dictated by the acceptor side-chain. Compared with PFSDCN, PFDCN with the additional conjugated –C=C– bond in the acceptor side-chain has a lower LUMO energy level from −3.27 eV to −3.43 eV and a reduced optical band gap from 2.05 eV to 1.87 eV.

High-Performance-Liquid-Chromatography (HPLC) grade chlorobenzene was purchased from Sigma-Aldrich (St. Louis, MO, USA) and was used as received. The sample solutions (9.0 × 10^−6^ M) were stirred in the dark for 12 h in a glove box filled with argon (oxygen concentration below 0.1 ppm) at room temperature. UV-visible absorption and fluorescence spectra, respectively were recorded on a Cary 50 absorption spectrometer (Varian Inc., Palo Alto, CA, USA) and a LS-55 fluorescence spectrometer (Perkin-Elmer, Waltham, MA, USA). Fluorescence quantum yields (Ф_F_) were determined following Demas and Grosby’s method [33] with the methanol solution of 3,3′-diethylthiadicarbocyanine iodide(DTDCI) as a reference (Ф_F_ = 0.36 ± 0.05) [34].

The visible and near-infrared femtosecond transient absorption (TA) spectrometer with a detection sensitivity better than 10^−4^ (change of optical density, ∆OD) is described below [17,35]. An optical parametric amplifier (OPA-800 CF-1, Spectra Physics, Mountain View, CA, USA) pumped by a regenerative amplifier (SPTF-100F-1KHPR, Spectra Physics) provided the actinic laser pulses at the desired wavelengths (~120 fs, full width at half maximum; fwhm). A white-light continuum probe was generated from a 3-mm-thick sapphire plate and was detected, after interrogating the excited sample (optical path length, 1 mm), with a liquid-nitrogen-cooled charge-coupled device detector (Spec-10:400B/LN) (Visible region λ = 430–700 nm) and an InGaAs linear array detector (OMA-V, Princeton Instruments, Trenton, NJ, USA) (Near infrared region λ = 830–1550 nm) attached to an imaging spectrograph (SpectraPro 2300i, Princeton Instruments). To ensure that each laser shot excited a fresh sample, the laser source was run at a repetition rate of 100 Hz (Spec-10:400B/LN be used to detected) and 333 Hz (OMA-V be used to detected). A mechanical chopper (Model 75158, Newport) was set in the pump beam to regulate pump “on” and “off” for a pair of sequential actinic pulses. The magic-angle scheme was used for the pump-probe measurement. The cross-correlation trace between the pump and the probe pulses was taken as the instrumental response function (IRF), and the temporal resolution was 160 fs as the fwhm of IRF. The diameter of the pump light on the sample is about 500 μm. A laser excitation fluence of 31 μJ/cm^2^ (at 400 nm) was used, corresponding to 6.30 × 10^13^ photons·cm^−2^·pulse^−1^. The time-resolved absorption spectra were corrected against group velocity dispersion.

## 4. Conclusions

The excited state dynamics of 2D-like conjugated copolymers PFDCN and PFSDCN in solution have been detected by femtosecond time-resolved absorption spectroscopy. The primary photoexcitations of PF(S)DCN solutions are mainly polaron pairs, which then self-localize and evolve to bound singlet exciton states within about 3–7 ps. The additional conjugated –C=C– bond in the side chain causes PFDCN having stronger intramolecular charge-transfer nature, therefore the photogeneration of polaron pairs of PFDCN has longer electron-hole distances and lower Coulombic attraction, which exhibits a longer self-localization time of 7 ps and shorter fluorescence lifetime of 1.48 ns. With respect to PFDCN, the photogeneration of polaron pairs of PFSDCN exhibits a shorter self-localization time of 3 ps and longer fluorescence lifetime of 1.60 ns. In addition, the conversion rate from polaron pairs to singlet exciton is 100% for PFDCN, while 54% for PFSDCN. The other 46% of polaron pairs in PFSDCN is lost by geminate recombine to the ground states. The occurrence of competition between polaron pairs-to-singlet exciton or polaron pairs-to-the ground state may reduce the excitation efficiency and ultimately affect *J*_SC_ of PSCs. These results providing more detailed insights into photophysical properties may promote the development of 2D-like copolymer photovoltaic devices. In future, the effects of excitation wavelength and intensity on the rate of photogenerated polaron pairs can be researched, which can increase the knowledge about copolymers.

## Figures and Tables

**Table 1:** Decay time constants (τ) derived from multi-exponential curve fitting of the kinetics for PFDCN chlorobenzene solution under excitation wavelengths at 400 nm (cf. Figure 3.)

Probe Wavelength	Fitting Parameter and Apparent Lifetime					
	a_1_	τ_1_ (ps)	a_2_	τ_2_ (ps)	a_3_	τ_3_ (ps)
530 nm	−1.05 × 10^−^^3^	2.57 ± 0.31	−0.49 × 10^−^^3^	16.44 ± 4.15	−2.84 × 10^−^^3^	1452 ± 16
630 nm			−11.19 × 10^−^^3^	6.99 ± 0.14	14.19 × 10^−^^3^	1476 ± 16
850 nm			5.68 × 10^−^^3^	7.42 ± 0.21	−1.66 × 10^−^^3^	1436 ± 73
1250 nm	−3.96 × 10^−^^3^	0.38 ± 0.03	−0.98 × 10^−^^3^	7.83 ± 0.40	3.87 × 10^−^^3^	1456 ± 26
1480 nm	−2.77 × 10^−^^3^	0.48 ± 0.04	−1.42 × 10^−^^3^	6.78 ± 0.85	4.33 × 10^−^^3^	1467 ± 34

**Table 2:** Decay time constants (τ) derived from multi-exponential curve fitting of the kinetics for PFSDCN chlorobenzene solution under excitation wavelengths at 400 nm (cf. Figure 5.)

Probe Wavelength	Fitting Parameter and Apparent Lifetime			
	a_1_	τ_1_ (ps)	a_2_	τ_2_ (ps)
520 nm	−4.49 × 10^−^^3^	3.28 ± 0.18	0.66 × 10^−^^3^	1037 ± 116
580 nm	−11.33 × 10^−^^3^	3.30 ± 0.10	12.86 × 10^−^^3^	1597 ± 20
700 nm	9.46 × 10^−^^3^	2.81 ± 0.37	−3.52 × 10^−^^3^	1499 ± 317
1250 nm	−1.27 × 10^−^^3^	3.83 ± 0.99	3.77 × 10^−^^3^	1581 ± 93

## Supplementary Materials

Supplementary Materials are available online.
