# Supplementary material for: Acceptor Side-Chain Effects on the Excited State Dynamics of Two-Dimensional-Like Conjugated Copolymers in Solution
Source: Molecules. 2017 Aug 25;22(9):1398. doi: 10.3390/molecules22091398 (PMC6151795; doi:10.3390/molecules22091398)
Supplement: Supplementary file 1 [file molecules-22-01398-s001.pdf]

Supplementary material

The effect of side chain on excited state dynamics in two-dimensional like conjugated copolymers in solution.

Ming-Ming Huo*,[a], Rong Hu[b], Wei Yan[a], Yi-Tong Wang[a,c], Kuan. W. A. Chee[a,c], Yong Wang[a], Jian-Ping Zhang[d]

[a], Qingdao Research Center for Advanced Photonics Technology, Laser Institute, Shandong Academy of Sciences, Qingdao 266100, China;

[b], Research Institute for New Materials Technology, Chongqing University of Arts and Sciences, Chongqing 402160, China;

[c], Department of Electrical and Electronic Engineering, Faculty of Science and Engineering, University of Nottingham Ningbo China, Ningbo 315100, China.

[d], Department of Chemistry, Renmin University of China, Beijing 100872, China

Supporting Figures and Tables

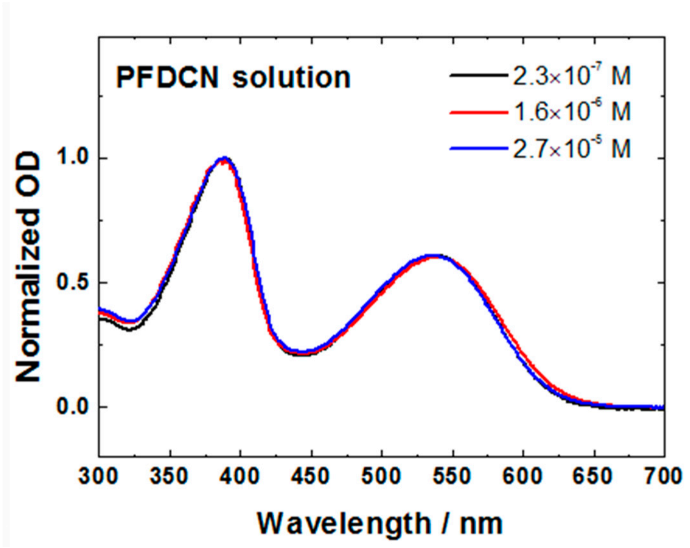

Figure S1. Normalized UV-visible absorption spectra of the chlorobenzene solutions of PFDCN at the indicated concentrations (based on monomer unit).

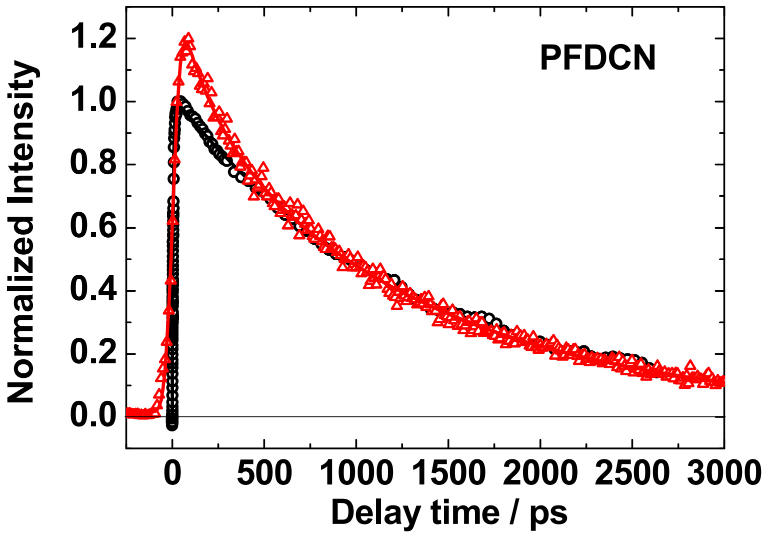

Figure S2. Comparison of the transient absorption (black) and the fluorescence (red) kinetics. The transient absorption kinetics for PFDCN chlorobenzene solution was plotted at 630 nm directly from the time-resolved absorption data under the photoexcitation at 400 nm, and the fluorescence kinetics were plotted at 700 nm are scaled arbitarily to the absorption kinetics. A slightly different between the sbsorption and fluorescence kinetics are due to the different excitation power.

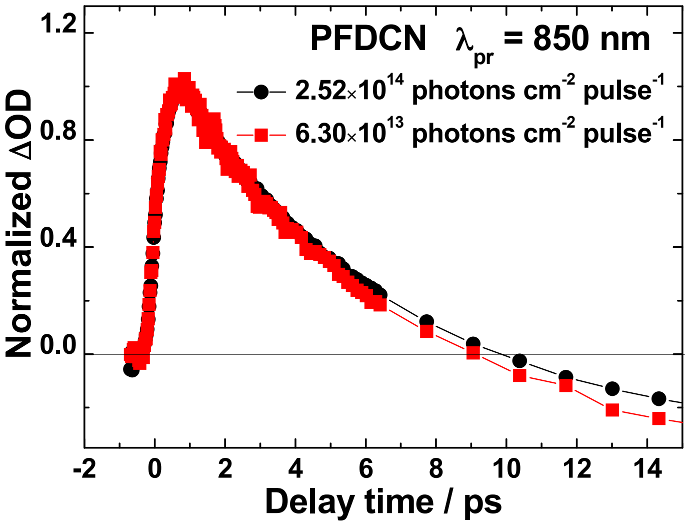

Figure S3. Kinetics of the 850 nm of PFDCN solution under high (black circle) and low (red square) excitation photons density. The excitation wavelength is 400 nm. The decay dynamics are independent of the excitation photons density in the first 8 ps.

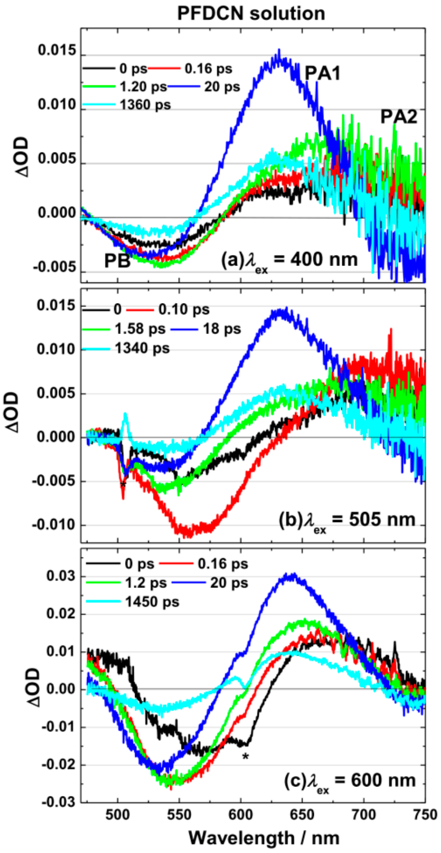

Figure. S4: fs-TA spectra of PFDCN solution with different excitation wavelength (a) λ_{ex} = 400 nm, (b) λ_{ex} = 505 nm, (c) λ_{ex} = 600 nm. The * are from the interference of the excitation light in (b) and (c). The photon energy of 3.1 eV (400 nm) is near the π-π* transition energy of the PFDCN conjugated main chain, whereas that of 2.45 eV (505 nm) is near the π-π* transition energy with

intramolecular charge transfer (ICT) characteristics, that of 2.07 eV (600 nm) is near the optical band-gap energy of the copolymer (1.87 eV). The existence of polaron pairs (PA2) is independent of excitaiton wavelength. However, the generation efficiency of polaron pairs may be influenced by the excitation wavelength and pulse energy.

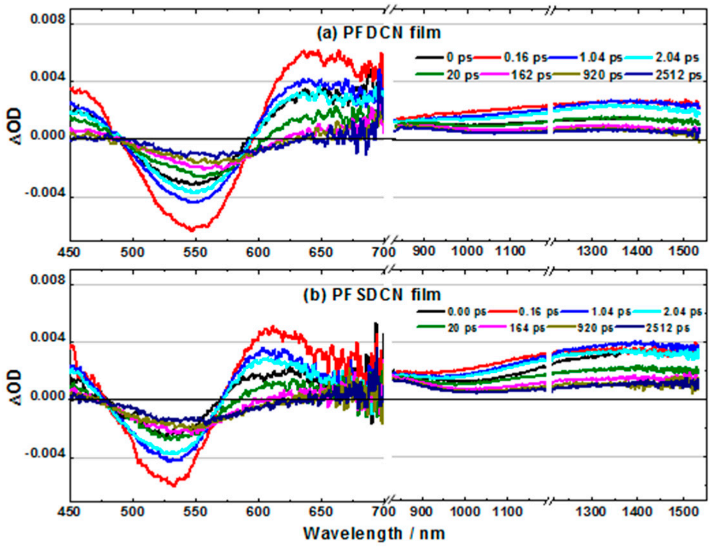

Figure S5: Transient absorption spectra of (a) PFDCN film and (b) PFSDCN film with excitation wavelength at 400 nm (3.65×10^{14} photons·cm^{-2}·pulse^{-1}). The break (//) from 700 nm to 830 nm, 1186 nm to 1216 nm where be interference by laser at 800 nm and 1200 nm (third order diffracted light of 400 nm).

In film preparation, neat PFDCN or neat PFSDCN (4 mg ml^{-1}) were dissolved in chlorobenzene solution. Quartz substrates were treated by ultrasonication in detergent and washed successively with deionized water, acetone, ethanol, and isopropyl alcohol, and on which neat PFDCN or neat PFSDCN film were spin-coated (600 rpm; 30 s). The preparations were conducted in a glovebox filled with argon (oxygen concentration <0.1 ppm).

The visible and near-infrared TA spectra of neat PFDCN film (Fig.S5a) and PFSDCN (Fig.S6b) film at 400 nm are shown in Fig. 1. Similar as PFDCN or PFSDCN solution, the negative feature of the TA spectra comes from photobleaching of ground state (PB). the absorption band around 630 nm (PFDCN film) and 600 nm (PFSDCN film) is originated from the $S_1 \rightarrow S_n$ singlet exciton. Different from the copolymer solution, the $S_1 \rightarrow S_n$ singlet exciton in the copolymer film appears immediately after the pulsed optical excitation, which means the increased intermolecular interactions in film may promote self-localization process of electrons and holes, shortening the geminate recombination time of Polaron Pairs→S_1. The wide absorption band around 1350 nm in Fig.1a and Fig.1b is from the absorption of polaron($P^{\bullet+}$) , because it is consistent with $P^{\bullet+}$ absorption spectrum obtained by spectroelectrochemical (SEC) spectra. Although the attribution of the 850 nm absorption band is not determined, according to its longer lifetime (>2.5 ns), it may be from the aborption of triplet state extion. Similar as the Diketopyrrolopyrrole film system, the triplet state absorption does not appear in Diketopyrrolopyrrole solution, but the triplet state are from the charge recombination in Diketopyrrolopyrrole film. [*[Williams, R. M.; Chen, H. C.; Nuzzo, D. D.; Meskers, S. D. J.; Janssen, R. A. J. Ultrafast Charge and Triplet State Formation in*

Diketopyrrolopyrrole Low Band Gap Polymer/Fullerene Blends:Influence of Nanoscale Morphology of Organic Photovoltaic Materials on Charge Recombination to the Triplet State. Journal of Spectroscopy, 2017,Article 6867507]]The results need to be further verified by ns-TA experiment or a SVD analysis.
